# The effects of oral health and social support on health-related quality of life of migrant older with children in Weifang, China

**DOI:** 10.1186/s12889-022-13843-0

**Published:** 2022-08-06

**Authors:** Jieru Wang, Jinfeng Zhao, Tingting Tian, Xiaoxu Jiang, Hexian Li, Mingli Pang, Fanlei Kong

**Affiliations:** 1 Centre for Health Management and Policy Research, School of Public Health, Cheeloo College of Medicine, Shandong University, Jinan, 250012 China; 2 NHC Key Lab of Health Economics and Policy Research, Shandong University, Jinan, 250012 China

**Keywords:** Migrant, Older adults, Health-related quality of life, Oral health, Social support, Observational study

## Abstract

**Background:**

With the accelerated urbanization and aging population in China, more and more migrant older with children (MOC) moved to new cities. Previous studies mainly explored the acculturation of MOC, yet few focused on the health conditions of this vulnerable group. This study aimed to investigate the effects of oral health and social support on health-related quality of life (HRQOL) of MOC in Weifang, China.

**Method:**

This study was a cross-sectional study and participants were selected by multi-stage cluster random sampling in Weifang, China. The HRQOL was assessed via the 12-item Short-Form Health Survey (SF-12) which included the mental component summary (MCS) and the physical component summary (PCS). The oral health was evaluated by the Geriatric Oral Health Assessment Index (GOHAI). The social support was administered using the Social Support Rating Scale (SSRS). Descriptive analysis was used to describe participants’ sociodemographic variables, oral health and social support. Univariate analysis and binary logistic regression analysis was used to investigate the association between the social support, oral health and HRQOL.

**Results and discussion:**

It was found that 25.0% of MOC were defined as MCS poor and PCS poor, respectively. Those participants with average and low monthly household income compared to those around them, average and poor oral health, and low levels of social support were more likely to have poor PCS. Those with temporary residence permits, fair and poor oral health, and medium and low levels of social support were more likely to report poor MCS.

**Conclusion:**

Results indicated that better social support and oral health led to higher HRQOL of MOC. Implications for the government, communities and families of MOC were given to improve their HRQOL.

**Supplementary Information:**

The online version contains supplementary material available at 10.1186/s12889-022-13843-0.

## Introduction

Since the reforming and opening up in 1978, China's economy has developed rapidly. Uneven economic development and rapid urbanization have led to population migration inside the country. According to the data of the seventh national census of China, the migrant population in China was about 376 million. Among them, the inter-provincial migrant older adults was about 125 million, and the intra-provincial migrant older adults was about 251 million [1]. Meanwhile, China has faced the problem of an aging population since the 1990s. Currently, there were about 264 million people aged 60 and above in China, accounting for 18.70% of the whole population, of which about 191 million people aged 65 and above, accounting for 13.50% [2].

With the development of China, the migrant population showed a trend of family migration, mainly manifested in the increasing proportion of children and older adults among the migrant older adults [3]. The migrant older adults has become a unique product of China's social transformation. The size of the older migrant adults increased from 5.03 million in 2000 to 13.04 million in 2015, with an average annual growth rate of 6.6% [1]. Among them, those older adults who leaved their hometown and migrated to big cities with their children was defined as migrant older with children (MOC) [4]. The arrival of MOC in cities brought new challenges not only to their health, but also to the public health. For example, MOC didn’t enjoy the public benefits as the local older people, unequally access to basic public health services, and the inability to pay high medical costs [5]. In addition to having an impact on their physical health, it could also affect their mental health to some extent. For example, older migrants may have language barriers that lead to poor communication with locals, which affected their social interactions and ultimately have an impact on their social support [6]. Therefore, the health of the MOC was an issue of social concern [7].

Health-related quality of life(HRQOL)was defined as the state of health of an individual under the influence of injuries, medical interventions, aging, and changes in the social environment, as well as subjective satisfaction linked to his or her economic and cultural background and values, and was considered a comprehensive assessment tool for the health status of a population. HRQOL was poorer for people with diabetes in rural China [8]. Another study of Chinese migrant workers to Korea [9] found that their physical health was similar to that of the locals while their mental health was lower. Studies on Chinese rural and urban migrant workers have shown lower HRQOL and higher prevalence compared to Chinese urban migrant workers [10]. Lu et al. [11] also concluded that the quality of life of Chinese older people who migrated with children was poor. However, relatively few researches has been done on the HRQOL of MOC. A study conducted in Jinan City, Shandong Province, China found that 25.2% and 25.0% of MOC were defined as poor MCS and poor PCS, respectively [7].

The HRQOL conceptual model was proposed by Wilson and Cleary which emphasized the importance of biological and physical factors for quality of life [12]. The World Health Organization(WHO) [13]defined oral health as a state free from oral and facial pain, oral diseases and disorders that limit an individual's ability to bite, chew, smile, speak, and psycho-social well-being. A previous study in the United States [14] showed that oral health was worse among minority or low-income older adults. These studies all suggested that older adults have poor oral health and needed more oral health care services [15]. And according to the 4^th^ national oral health epidemiological survey, the oral problems of Chinese older people were even more serious [16]. A study on the oral health status of the mainland Chinese people [17] found that oral health deteriorated with age. As one of the components of overall health, oral health was critical to both overall health and well-being and greatly affected quality of life among the Chinese old adults [18]. By negatively affecting physical, psychological and social aspects, further development of oral diseases could cause a decrease in the ability to perform daily life, which ultimately affected people's quality of life through its impact on their daily lives. Ding Hui et al. [19] concluded that the oral health status of older adults over 60 years of age was poor in rural Yunnan of China, and the oral diseases affected the quality of life of older adults. However, there was no research on the impact of oral health status on the HRQOL of MOC in China. Therefore, it was hypothesized that oral health would be positively related to HRQOL of MOC.

In addition, the HRQOL conceptual model emphasized the importance of social support for quality of life [12]. Social support referred to communication and connection between individuals and others for information and comfort [20]. It was generally a general term for the various kinds of support that come from outside the individual, and was the social behavior that accompanies the existence of a vulnerable group. In Korea [21], social support was an important factor contributing to the HRQOL domain of MCS scores of older adults living alone. Cheng Gang et al. [22], on the other hand, found that there were gender and residence differences in social support on the life satisfaction of Chinese older adults, thus affecting their well-being and quality of life. Another study found that older adults without children have lower levels of social support, which could further led to disadvantages in terms of well-being and health [23].Thus, it was also hypothesized that social support would be positively related to HRQOL of MOC.

In summary, few studies that included MOC as their study population in China, and none study had examined the effects of oral health status and social support on HRQOL of MOC. Thus, this study aimed to investigate the effects of oral health status and social support on HRQOL in the MOC in Weifang, China.

## Method

### Data and sample

This was a cross-sectional study and the data were collected in August 2021 in Weifang City, Shandong Province, China. As of 2018, the city has four districts, two counties and six county-level cities under its jurisdiction, with a total area of 16,167.23 square kilometers. According to the results of the seventh national census, as of 00:00 on November 1, 2020, the resident population of Weifang City was approximately 9.3 million, and at the end of the year, the city's registered population was about 9.31 million [24]. In this study, people aged 60 years or older who came to Weifang with their children were used as the study population. A multi-stage cluster random sampling method was used to select participants.

In the first stage of data collection, considering the influence of economic development and geograph1ical location, 4 of the 12 subordinate districts in Weifang were selected as the main sampling units in urban areas, which would have better economic development and be more attractive to the migrant population compared with other districts. In the second stage, four streets also were selected randomly from these four areas as sub-sampling units. In the third stage, four communities were selected from each subsampling unit. In these communities, all migrant older aged 60 years or older who came to Weifang with their children constituted the total sample for this study. The inclusion criteria for participants were (1) aged 60 or older, (2) non-local residents in the household registration area, and (3) able to understand Mandarin and communicate smoothly with the surveyors.

The investigators for this study consisted of 25 college students. Before the survey began, these investigators were trained in the following areas: research background, questionnaire content, social survey skills, etc. The investigators collected data by conducting approximately 30 min of face-to-face interviews with survey respondents. Prior to the survey, the investigators would ask for the consent of the respondents before conducting face-to-face interviews. Therefore, the questionnaire return rate of the study was 100%.

### Measurement

#### Social demographic characteristics

Data were collected through a questionnaire consisting of three scales measuring quality of life, oral health status and social support, as well as demographic characteristics. The sociodemographic characteristics included sex, age, *hukou*, marriage status, education level, have any income, source of living expenses, migrant willing, type of accompanying space, temporary residence permit and comparison of monthly household income with others around. All of the measurements (and the corresponding options) of the variables were included in Supplementary Table 1.

#### Oral health status

The Chinese version of the Geriatric Oral Health Assessment Index (GOHAI) [25, 26] was used to measure oral health status. The GOHAI was originally developed to assess the self-reported oral health status of older adults and has been widely used in China and abroad. The Chinese version of GOHAI was divided into three sub-dimensions and 12 items designed to assess different aspects of oral health, namely (1) physical functioning, including eating, speaking, and swallowing; (2) psychosocial functioning, including concerns or worries about oral health, dissatisfaction with appearance, self-awareness of oral health, and avoidance of social contact due to oral problems; and (3) pain or discomfort, including use of medications to relieve oral pain or discomfort. Higher GOHAI scores indicated better perceived oral health status. The GOHAI scores was divided into three categories: those below 50 were defined as having poor oral health; 51–56 as having average oral health; and 57–60 as having good oral health [26]. The GOHAI also had good reliability (Cronbach's alpha coefficient of 0.874) and validity in the present study.

#### Social support

This study used the Social Support Rating Scale (SSRS) [20] to measure the social support of MOC, including 10 types of support: friends, residents, neighbors, colleagues, family members, financial, comfort, conversation, help, and activities. The social support scale has ten items, including three dimensions of objective support (3 items), subjective support (4 items), and utilization of social support (3 items). The SSRS has been proven to have good reliability and validity in practice, and has been widely used in China. A higher total social support score meant that the subject received more social support. The range of the total score of the scale is 0–66. It could be divided into three categories: (1) total score ≤ 22 was low level social support; (2) 23 ≤ total score ≤ 44 was medium level social support; (3) 45 ≤ total score ≤ 66 was high level social support [27].

#### Health-related quality of life

This study used a brief 12-item health survey (SF-12) [25, 28] to measure the HRQOL of the sample. The SF-12 was a self-reported outcome indicator used to assess the impact of health on an individual's daily life. It often used as a measure of quality of life and was a shortened version of its predecessor, the SF-36, which itself evolved from the Medical Outcomes Study. The Chinese version of SF12 was practically tested to have good reliability (Cronbach's a = 0.910) and validity (Spearman's *r* = 0.730, *p* < 0.05) [29]. Currently, SF12 was widely used in the practice of measuring HRQOL in China and abroad. The SF12 consisted of 12 items and could be divided into two subscales: Physical Component Summary (PCS) and Mental Component Summary (MCS), which were used to measure physical and mental HRQOL, respectively. The PCS and MCS scores ranged from 0 ~ 100, with higher scores indicating better health status. In this study, the quartiles of PCS and MCS were calculated and dichotomized them using the cut-off point of the first quartile of their scores; scores below the first quartile were defined as poor HRQOL and the remaining three quartiles as good HRQOL [30–32].

### Statistics analysis

All statistical analyses were performed with SPSS 22.0, and *P* values < 0.05 were considered statistically significant differences. Firstly, the chi-square tests was used to explore the difference between the related variables and HRQOL. Following a previous research, *P* values < 0.2 was applied as a criterion for selecting the variables in the logistics regression analysis [33]. Since the PCS and MCS were both dichotomous variables, a binary logistic regression analysis was chosen [34]. In this study, a total of three dichotomous logistic regression models were developed by including three types of variables, socio-demographic characteristics, social support, and oral health, while odds ratios (or) and 95% confidence intervals (95% ci) were calculated.

## Results

### Participants characteristic

A total of 613 participants were enrolled in the study. Table 1 provided basic information on the sociodemographic characteristics, oral health status, and social support of the 613 participants. The results showed that the majority of the sample in this study was female (73.1%), 486 of the sample were between 60 and 69 years of age (79.3%), and 539 of the sample were married (87.9%). The overall education level of the respondents in this study was relatively low, including 161 participants of illiteracy and185 people with elementary school education, and only 109 people with high school education and above. 70.8% of MOC had no income; living expenses came mainly from their spouses and children (393, 64.0%). In addition, 82.4% of MOC did not have a temporary residence permit in the place where they migrated. In terms of willingness to move with them, only a minority of the sample was neutral and did not want to move with them (101, 16.5%). In addition, in terms of the spatial type of accompanying migration, most of them migrated across districts/counties (430, 70.1%), and only 53 migrated across provinces (8.7%).

**Table 1:** Sociodemographic characteristics and descriptive analysis of MOC

Variable	Total	Good PCS	Poor PCS	*P*	Good MCS	Poor MCS	*P*
	**N(%)**	**N(%)**	**N(%)**		**N(%)**	**N(%)**	
	613(100)	460(75.0)	153(25.0)		460(75.0)	153(25.0)	
**Sex**				0.085			0.702
Male	165(26.9)	132(80.0)	33(20.0)		122(73.9)	43(26.1)	
Female	448(73.1)	328(73.2)	120(26.8)		338(75.4)	110(24.6)	
**Age(year)**				0.001			0.913
60–69	486(79.3)	386(79.4)	100(20.6)		363(74.7)	123(25.3)	
70–69	103(16.8)	65(63.1)	38(36.9)		79(76.7)	24(23.3)	
80-	24(3.9)	9(37.5)	15(62.5)		18(75.0)	6(25.0)	
**Hukou**				0.017			0.112
Rural	525(85.6)	385(73.3)	140(26.7)		388(73.9)	137(26.1)	
Urban	88(14.4)	75(85.2)	13(14.8)		72(81.8)	16(18.9)	
**Marriage Status**				0.015			0.015
Married	539(87.9)	413(76.6)	126(23.4)		413(76.6)	126(23.4)	
Mateless	74(12.1)	47(63.5)	27(36.5)		47(63.5)	27(36.5)	
**Education**				0.004			0.155
Illiterate	161(26.3)	110(68.3)	51(31.7)		113(70.2)	48(29.8)	
Primary school	185(30.2)	132(71.4)	53(28.6)		149(80.5)	36(19.5)	
Junior high school	158(25.8)	124(78.5)	34(21.5)		116(73.4)	42(26.6)	
High school and above	109(17.7)	94(86.2)	15(13.8)		82(75.2)	27(24.8)	
**Have Any Income**				0.017			0.633
Yes	179(29.2)	146(81.6)	33(18.4)		132(73.7)	47(26.3)	
No	434(70.8)	314(72.4)	120(27.6)		328(75.6)	106(24.4)	
**Source of Living Expenses**				0.002			0.340
Own	214(34.9)	170(79.4)	44(20.6)		162(75.7)	52(24.3)	
Spouse and children	393(64.0)	289(73.5)	104(26.4)		292(74.3)	101(25.7)	
Others	6(0.1)	1(16.7)	5(83.3)		6(100.0)	0(0.0)	
**Migration Willing**				0.619			0.037
Reluctant	56(9.2)	39(69.6)	17(30.4)		35(62.5)	21(37.5)	
Neutral	45(7.3)	34(75.6)	11(24.4)		31(68.9)	14(31.1)	
Willing	512(83.5)	387(75.6)	125(24.4)		394(77.0)	118(23.0)	
**Type of Accompanying Space**				0.681			0.012
Cross-district/county	430(70.1)	323(75.1)	107(24.9)		337(78.4)	93(21.6)	
Cross prefecture level cities	130(21.2)	95(73.1)	35(26.9)		86(66.2)	44(33.8)	
Cross-provincial	53(8.7)	42(79.2)	11(20.8)		37(69.8)	16(30.2)	
**Temporary Residence Permit**				0.217			0.007
With	108(17.6)	76(70.4)	32(29.6)		70(64.8)	38(35.2)	
Without	505(82.4)	384(76.0)	121(24.0)		390(77.2)	115(22.8)	
**Comparison of Monthly Household Income with Others Around**				0.001			0.001
Higher	66(10.8)	61(92.4)	5(7.6)		48(72.7)	18(27.3)	
Similar	265(43.2)	202(76.2)	63(23.8)		220(83.0)	45(17.0)	
Lower	282(46.0)	197(69.9)	85(30.1)		192(67.4)	90(32.6)	
**Oral Health**				0.001			0.001
Poor	118(19.3)	64(54.2)	54(45.8)		70(59.3)	48(40.7)	
General	151(24.6)	107(70.7)	44(29.3)		109(72.2)	42(27.8)	
Good	344(56.1)	289(84.0)	55(16.0)		281(81.7)	63(18.3)	
**Social Support**				0.001			0.001
Poor	5(0.9)	2(40.0)	3(60.0)		2(40.0)	3(60.0)	
General	468(76.3)	337(72.0)	131(28.0)		337(72.0)	131(28.0)	
Good	140(22.8)	121(86.4)	19(13.6)		121(86.4)	19(13.6)	

*MOC* Migrant older with children, *MCS* Mental component summary, *PCS* Physical component summary

### HRQOL in the mental and physical dimensions

As shown in Table 1, the percentage of both poor PCS and poor MCS were 25%. Factors that differed significantly in PCS included sex (*P* < 0.1), age (*P* < 0.001), *hukou* (*P* < 0.05), marital status (*P* < 0.05), education level (*P* < 0.01), presence of income (*P* < 0.05), main source of living expenses (*P* < 0.01), and high monthly household income compared to those around them (*P* < 0.001). Factors that differed significantly in MCS included *hukou* (*P* < 0.2), marital status (*P* < 0.05), education level (*P* < 0.2), willingness of migrant (*P* < 0.05), type of space to migrate (*P* < 0.05), presence of temporary residence permit (*P* < 0.01), and higher monthly household income compared to those around them (*P* < 0.001).

### Relationship between independent variables and HRQOL in the physical domains

Tables 2 and 3 show the OR, and 95% CI of the associations of statistically significant variables after univariate analysis for PCS and MCS, respectively.

**Table 2:** The binomial logistic regression of demographic characteristics, oral health and social support of PCS

Variable	Model 1		Model 2		Model 3	
	**Demography**		**Model 1 + GOHAI**		**Model 2 + Social Support**	
	**OR**	**95%CI**	**OR**	**95%CI**	**OR**	**95%CI**
**Sex**						
Female	1.0		1.0		1.0	
Male	0.731	0.431–1.240	0.732	0.427–1.256	0.729	0.422–1.259
**Age(year)**						
80-	1.0		1.0		1.0	
70–69	0.116	0.043–0.313	0.146	0.053–0.405	0.147	0.053–0.409
60–69	0.278	0.103–0.752	0.319	0.114–0.893	0.319	0.113–0.900
**Hukou**						
Urban	1.0		1.0		1.0	
Rural	1.815	0.852–3.992	1.596	0.718–3.548	1.481	0.660–3.322
**Marriage Status**						
Mateless	1.0		1.0		1.0	
Married	1.113	0.602–2.055	1.127	0.601–2.113	1.307	0.679–2.516
**Education**						
Illiterate	1.0		1.0		1.0	
Primary school	0.951	0.579–1.563	0.936	0.560–1.562	0.992	0.591–1.664
Junior high school	0.844	0.476–1.495	0.902	0.502–1.621	0.955	0.528–1.730
High school and above	0.678	0.324–1.419	0.782	0.370–1.654	0.843	0.396–1.797
**Have Any Income**						
Yes	1.0		1.0		1.0	
No	1.072	0.571–2.013	1.167	0.610–2.232	1.215	0.629–2.347
**Source of Living Expenses**						
Others	1.0		1.0		1.0	
Spouse and children	0.103	0.011–0.962	0.105	0.011–1.019	0.100	0.010–1.013
Own	0.087	0.010–0.722	0.079	0.008–0.714	0.075	0.008–0.722
**Comparison of Monthly Household Income with Others Around**						
Higher	1.0		1.0		1.0	
Similar	4.110	1.494–11.304	3.835	1.364–10.780	3.729	1.319–10.541
Lower	3.004	1.103–8.184	2.909	1.046–8.088	2.817	1.008–7.870
**Oral Health**						
Good			1.0		1.0	
General			3.573	2.179–5.860	3.381	2.055–5.563
Poor			1.920	1.191–3.548	1.883	1.162–3.052
**Social Support**						
Good					1.0	
General					5.186	0.654–41.153
Poor					1.921	1.096–3.366

*PCS* Physical component summary, *MOC* Migrant older with children, *OR* Crude odds ratios, *95% CI* 95% confidence intervals

**Table 3:** The binomial logistic regression of demographic characteristics, oral health and social support of MCS

Variable	Model 1		Model 2			Model 3	
	**Demography**		**Model 1 + GOHAI**			**Model 2 + Social Support**	
	**OR**	**95%CI**	**OR**	**95%CI**		**OR**	**95%CI**
***Hukou***							
Urban	1.0		1.0			1.0	
Rural	2.114	1.059–4.211	1.991	0.984–4.031		1.901	0.934–3.867
**Marriage Status**							
Mateless	1.0		1.0			1.0	
Married	0.554	0.321–0.956	0.584	0.335–1.020		0.658	0.368–1.175
**Education**							
Illiterate	1.0		1.0			1.0	
Primary school	0.605	0.386–1.095	0.632	0.371–1.077		0.673	0.394–1.152
Junior high school	1.005	0.599–1.686	1.112	0.654–1.890		1.167	0.683–1.993
High school and above	1.079	0.583–1.999	1.270	0.675–2.388		1.390	0.734–2.633
**Comparison of Monthly Household Income with Others Around**							
Higher	1.0		1.0			1.0	
Similar	1.068	0.537–2.122	0.971	0.586–2.138		0.935	0.461–1.894
Lower	0.456	0.228–0.913	0.414	0.204–0.840		0.393	0.193–0.803
**Temporary Residence Permit**							
Without	1.0		1.0			1.0	
With	2.255	1.394–3.647	2.114	1.289–3.465		1.943	1.179–3.203
**Type of Accompanying Space**							
Cross- provincial	1.0		1.0			1.0	
Cross prefecture level cities	0.526	0.226–1.041	0.551	0.274–1.110		0.587	0.290–1.187
Cross- district/county	1.021	0.486–2.146	1.133	0.530–2.422		1.185	0.552–2.546
**Migration Willing**							
Willing	1.0		1.0			1.0	
Neutral	2.109	1.135–3.918	2.083		1.100–3.942	2.006	1.055–3.815
Reluctant	1.479	0.734–2.979	1.566		0.760–3.224	1.506	0.729–3.142
**Oral Health**							
Good			1.0			1.0	
General			3.097		1.881–5.098	2.938	1.780–4.848
Poor			1.827		1.139–2.932	1.783	1.106–2.874
**Social Support**							
Good						1.0	
General						3.740	0.487–28.720
Poor						1.979	1.131–3.464

*MCS* Mental component summary, *MOC* Migrant older with children, *OR* Crude odds ratios, *95% CI* 95% confidence intervals

In Table 2, Model 1 contained only the socio-demographic variables. The results showed that age, main source of living expenses and comparison with the monthly household income of the surrounding people were significant factors for PCS. These three variables remained statistically significant when oral health were entered into Model 2. Oral health were also statistically significant in Model 2. Model 3 included sociodemographic variables, oral health status and social support. Model 3 showed that age, primary source of living expenses, comparison of monthly household income with those around them, oral health status and social support were statistically associated with PCS. Specifically, 60–69 (OR = 0.319, 95%CI = 0.113–0.900) and 70–79 MOC (OR = 0.147, 95%CI = 0.053–0.409), living expenses mainly depended on their own (OR = 0.075, 95%CI = 0.008–0.722) were more likely to have good PCS. However, for those MOC with similar household income compared to those around them (OR = 3.729, 95%CI = 1.319–10.541) as well as lower (OR = 2.817, 95%CI = 1.008–7.870), fair oral health (OR = 3.381, 95%CI = 2.055–5.563) as well as poor (OR = 1. 883, 95%CI = 1.162–3.052), and low level of social support (OR = 1.921, 95%CI = 1.096–3.366), these scores were reversed. Among them, the OR for poor oral health was 1.883, indicating that the degree of risk of poor PCS among MOC with good oral health was 1.883 times than that good oral health. The OR of low level social support was 1.921, indicating that the degree of risk of poor PCS was 1.921 times among MOC with low level social support than high level social support.

### Relationship between independent variables and HRQOL in the mental domains

As shown in Table 3, Model 1 contained sociodemographic variables, of which *hukou*, marital status, education level, presence of a temporary residence permit, willingness to migrate. With the inclusion of oral health status in Model 2, the significance of *hukou* and marital status disappeared, and oral health status was also statistically significant. Social support was added to Model 2 to constitute Model 3. The results of Model 3 showed that comparative monthly household income with the surrounding people, presence of temporary residence permit, willingness to migrate with the family, oral health status and social support were significantly associated with MCS. Specifically, the MOC with lower monthly household income (OR = 0.393, 95%CI = 0.193–0.803) than those around them were more likely to report good MCS. Conversely, MOC who had a temporary residence permit ( OR = 1.943, 95%CI = 1.179–3.203), were neutral in their willingness to migrate (OR = 2.006, 95%CI = 1.055–3.815), had fair (OR = 2.938, 95%CI = 1.780–4.848) and poor (OR = 1.783, 95%CI = 1.106–2.874) oral health status, and had low levels of social support (OR = 1.979, 95%CI = 1.131–3.464) were more likely to report poor MCS. Among them, the OR for poor oral health was 1.783, indicating that the degree of risk of poor MCS among MOC with good oral health was 1.783 times than that good oral health. The OR of low level social support was 1.979, indicating that the degree of risk of poor MCS was 1.979 times among MOC with low level social support than high level social support.

## Discussion

This study clarified the status of HRQOL among the MOC in Weifang, Shandong Province, China, which was beneficial to enrich the research about the MOC’s HRQOL. Moreover, this study also initially explored the relationship between oral health, social support and HRQOL, which could provide new empirical evidence for policy makers and HRQOL related researchers who aimed to improve the HRQOL of MOC. In this study, the mean PCS and MCS scores of the MOC were 47.51 ± 11.31 and 56.74 ± 7.7.40, respectively. Compared to older adults [35] in Chengdu City, Sichuan Province of China, both PCS and MCS were higher in Weifang City, China. This may be due to the fact that the MOC were able to live with their families and family reunification has a positive impact on the psychological health of the older migrants [36]. At the same time, the main reason for the older adults to migrate with their children was to take care of their grandchildren, which requires them to have a relatively good health condition.

The results of this study showed that three-quarters of MOC were classified as “good” on the PCS and MCS, the results was similar to Tian’s study [31]. This study showed that age, primary source of living expenses, and comparison with the monthly household income of the surrounding people were statistically significantly associated with the PCS of MOC in the sociodemographic factors.

Many studies have shown that age, one of the main factors affecting quality of life in older adults, was negatively associated with the PCS, which meant that their physical health may deteriorate as their age increased [37–39]. As the body ages, the organs and tissues of the older adults naturally age, organ function gradually declines, metabolic processes slow down, physical activity decreases, and overall health deteriorates [40].

Apart from that, the results of this study indicated that MOC whose living expenses were mainly derived from themselves, their spouses and children were positively associated with their PCS. This may be due to more stable and reliable living expenses from themselves, their spouses and children would generally result in a more secure life and a higher standard of living and quality of life for the MOC [41, 42]. Moreover, the seniors who migrated with children with lower monthly household incomes were more likely to have worse PCS than those around them. One of the reasons for this may be that the level of monthly household income directly affects the standard and quality of living [43]. With relatively low household income, their standard of living and quality of life may also be lower, which was detrimental to the health status of the MOC. In other words, the better the overall economic situation of MOC, the better their physical health is.

As for the MCS, the relevant determinants were marital status, presence of temporary residence permit, and willingness to move with the family. Among these, having a spouse was a protective factor for MCS in MOC. And MOC with a spouse were more likely to report good MCS. This was consistent with the results of previous studies. Previous research [44, 45] had shown that married older adults were less likely to suffer from mental illnesses (such as depression).

In contrast, having a temporary residence permit and being neutral in their willingness to migrate were statistically negative determinants for MCS among MOC. A possible reason for having a temporary residence permit but a poor MCS was that the permit would highlight the non-local identification of MOC, which may psychologically hurt their emotional feelings, thus making their MCS possibly worse [24]. This finding was not same as previous studies, since previous researches had found that new urban immigrants with residence permits felt more institutionalized acceptance than exclusion [46]. In addition, for the MOC who were neutral in their willingness to migrate (they were not entirely willing to leave their hometown), performed worse on the MCS [47]. The possible reason was that the emotional needs of MOC are more intense, and the temporary nature of their life in the city is detrimental to their mental health [48]. Previous studies [47–49] also support this view.

Previous studies have found that oral health has an impact on people’s HRQOL from childhood and continues into old [50, 51]. Previous findings found that poor oral health was a risk factor for HRQOL in older adults [52, 53]. Consistent with previous studies, our study found that MOC with poor oral health also had poorer PCS and MCS. A survey of older adults in the United States found a positive association between oral health and quality of life which was consistent with the results of this study [54]. The present study showed that oral health was significantly positively associated with both PCS and MCS in MOC. In the present study, MOC with fair and poor oral health were more likely to report poor PCS and MCS. Another study on the relationship between oral health and quality of life among older adults in southwest China also found that oral disease had a considerable negative impact on QOL in older adults [55]. Liu and Chu pointed that oral health was an important component of human health and quality of life, and was an indispensable pillar in extending a person’s healthy life span, therefore, maintaining oral health would be helpful to promote the health of the whole body, improve the quality of life and prolong the life span of people, and oral health promotes the health of the whole body [56].

Previous studies have demonstrated that social support was associated with HRQOL [57], the similar results was also found in the present study. Some researches suggested [58, 59] that spouses could provide stability and emotional comfort to immigrant older adults and may make an important contribution to the development of healthy behavioral habits. Also, the fact that living expenses mainly come from oneself, one’s spouse and children were found to be statistically significantly associated to the MCS. The family is the main source of dependence for MOC and living with family members is beneficial to their health. The majority of MOC in the survey sample lived with their families, so family support and care may have led to higher levels of health and life satisfaction, similar to the results of previous studies [60, 61]. Our study was similar to previous studies which also found the majority of MOC have a moderate level of social support [7]. This may be due to the fact that the subjects of this survey were MOC who mostly from rural areas and whose social support network in the city has not been fully constructed [62].

In order to improve the HRQOL of MOC, the following measures could be taken. First of all, since oral health status could affect HRQOL, it is necessary to continuously raise awareness of the importance of oral health among the older people. For example, the community could hold oral health education activities; on the other hand, the family members who lived with MOC could help them to develop the habit of daily teeth brushing. Secondly, because social support also had an important impact on HRQOL, so it is essential to ensure that MOC could get a high level of social support. This requires a concerted effort from all parties, as social support comes from a wide range of sources, including family, community, and society. Thirdly, the main sources of living expenses and the comparison of monthly household income with the surrounding population also play a role in the study. Considering the older age of MOC, the target of intervention could be shifted to their children, for example, the government creates a more equitable employment environment for young migrants, focuses on expanding employment channels, and increases the employment rate of MOC children.

### Limitations

This study has some limitations. Firstly, due to the impact of the COVID-19 pandemic, the survey could not be conducted in Shanghai as planned, and the data were limited to Weifang, so that may not be representative of other regions in China. Secondly, the instrument used to measure oral health in this study was the GOHAI, which focused mainly on the measurement of their subjective perceptions without examining the clinical aspects of participants. There is a lack of knowledge about the oral health status of the study population in terms of clinical aspects. In the future, clinical oral health will be conducted to enrich the research. Thirdly, this study is a cross-sectional study and therefore cannot predict the causal relationship between variables. Lastly, there might be a selection bias based on the sampling method of this study, which should be avoided as much as possible in the future studies.

## Conclusion

In summary, this is the first study which clarified the effect of oral health and social support on HRQOL in the Chinese MOC population. It was found that 25% of MOC reported poor PCS and MCS. Average and low monthly household income compared to those around them, fair and poor oral health, and low level of social support had a negative effect on PCS of the MOC. Having a temporary residence permit, having fair and poor oral health, and having a medium or low level of social support was negatively associated with MCS of the MOC. It is hoped that the results of this study could provide empirical reference for the improvement of health related quality of life and well-being of MOC.

## Supplementary Information

**Additional file 1: Supplementary Table 1.** Measurements of the variables in this study.

## Data Availability

Under reasonable requirements, the data and material of this study can be obtained from the corresponding author. The data are not publicly available due to privacy restrictions.

## Abbreviations

MOC: Migrant older with children
HRQOL: Health-related quality of life
MCS: Mental Component Summary
PCS: Physical Component Summary
OR: Odds ratio
CI: Confidence interval
