# Supplementary material for: The effects of oral health and social support on health-related quality of life of migrant older with children in Weifang, China
Source: BMC Public Health. 2022 Aug 6;22:1505. doi: 10.1186/s12889-022-13843-0 (PMC9357308; doi:10.1186/s12889-022-13843-0)
Supplement: Supplementary file 1 — Additional file 1: Supplementary Table 1. Measurements of the variables in this study. [file 12889_2022_13843_MOESM1_ESM.docx]

**The effects of oral health and social support on health-related quality of life of migrant older with children in Weifang, China**

Supplementary Table 1. Measurements of the variables in this study

| Variable | Question | Options |
| --- | --- | --- |
| Age | How old are you? |  |
| *Hukou* | Are you from an urban or rural area? | Urban |
|  |  | Rural |
| Marriage Status | Are you married? | Married, |
|  |  | Mateless |
| Education Level | What is your education level? | Illiterate |
|  |  | Primary school |
|  |  | Junior high school |
|  |  | High school and above |
| Have Any Income | Do you have income? | Yes |
|  |  | No |
| Source of Living Expenses | Who are your main sources of living expenses? | Own |
|  |  | Spouses and children |
|  |  | Others |
| Migration Willing | Would you like to immigrate? | Reluctant |
|  |  | Neutral |
|  |  | Willing |
| Type of Accompanying Space | What type of immigrant are you? | Cross-district/county |
|  |  | Cross prefecture level cities |
|  |  | Cross-provincial |
| Temporary Residence Permit | Do you have a temporary residence permit? | Yes |
|  |  | No |
| Comparison of Monthly Household Income with Others Around | What level of income do you think your family is at compared to others around you? | Higher |
|  |  | Similar |
|  |  | Lower |
